# Computer Simulation to Rationalize “Rational” Engineering of Glycoside Hydrolases and Glycosyltransferases

**DOI:** 10.1021/acs.jpcb.1c09536

**Published:** 2022-01-24

**Authors:** Joan Coines, Irene Cuxart, David Teze, Carme Rovira

**Affiliations:** † Departament de Química Inorgànica i Orgànica and Institut de Química Teòrica i Computacional (IQTCUB), Universitat de Barcelona, Barcelona 08028, Spain; ‡ The Novo Nordisk Foundation Center for Biosustainability, Technical University of Denmark, Kgs. Lyngby 2800, Denmark; § Institució Catalana de Recerca i Estudis Avançats (ICREA), Passeig Lluís Companys 23, Barcelona 08010, Spain

## Abstract

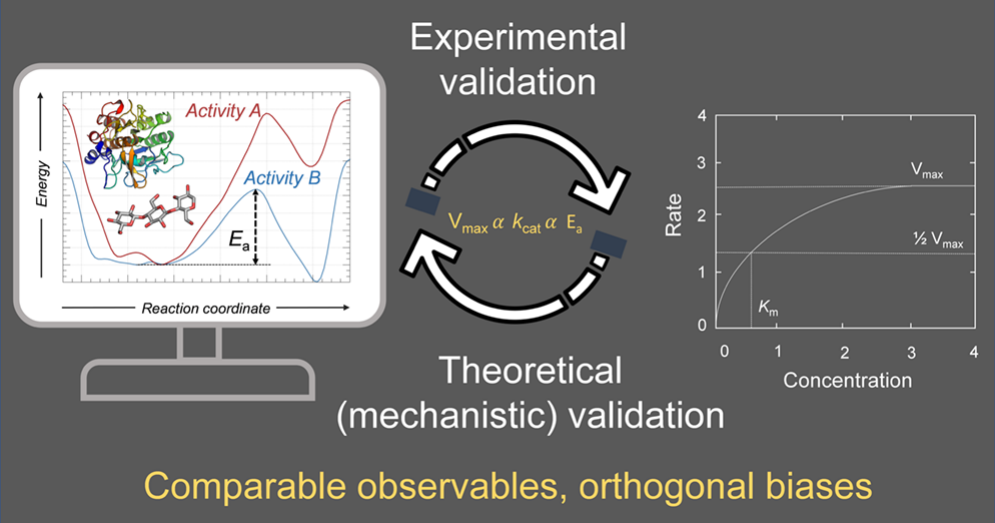

Glycoside hydrolases and glycosyltransferases are the main classes of enzymes that synthesize and degrade carbohydrates, molecules essential to life that are a challenge for classical chemistry. As such, considerable efforts have been made to engineer these enzymes and make them pliable to human needs, ranging from directed evolution to rational design, including mechanism engineering. Such endeavors fall short and are unreported in numerous cases, while even success is a necessary but not sufficient proof that the chemical rationale behind the design is correct. Here we review some of the recent work in CAZyme mechanism engineering, showing that computational simulations are instrumental to rationalize experimental data, providing mechanistic insight into how native and engineered CAZymes catalyze chemical reactions. We illustrate this with two recent studies in which (i) a glycoside hydrolase is converted into a glycoside phosphorylase and (ii) substrate specificity of a glycosyltransferase is engineered toward forming *O*-, *N*-, or *S*-glycosidic bonds.

## Introduction

Carbohydrates are ubiquitous in the biosphere, being involved in virtually all biological processes and constituting most of the biomass. Together with polynucleotides and polypeptides, carbohydrates encode much of the information transfer in biological systems.^1^ Understanding, control and modification of carbohydrate structures is often hampered by the intricacies of synthetic glycochemistry^2^ and the large diversity of carbohydrate structures. Bonds between carbohydrate monomers can exhibit various linkage positions and stereochemistries. Just considering the ten mammalian monosaccharides (e.g., α,β-glucose or α,β-mannose), more than 13 billion structures are possible for a “simple” tetrasaccharide.^1^ Branching can further increase the structural complexity of carbohydrates. Individual monosaccharides have been portrayed as letters of an alphabet (the third alphabet of life, after proteins and nucleic acids) that combine to form words of the “sugar code” or glycomics.^3^

**Scheme 1:**
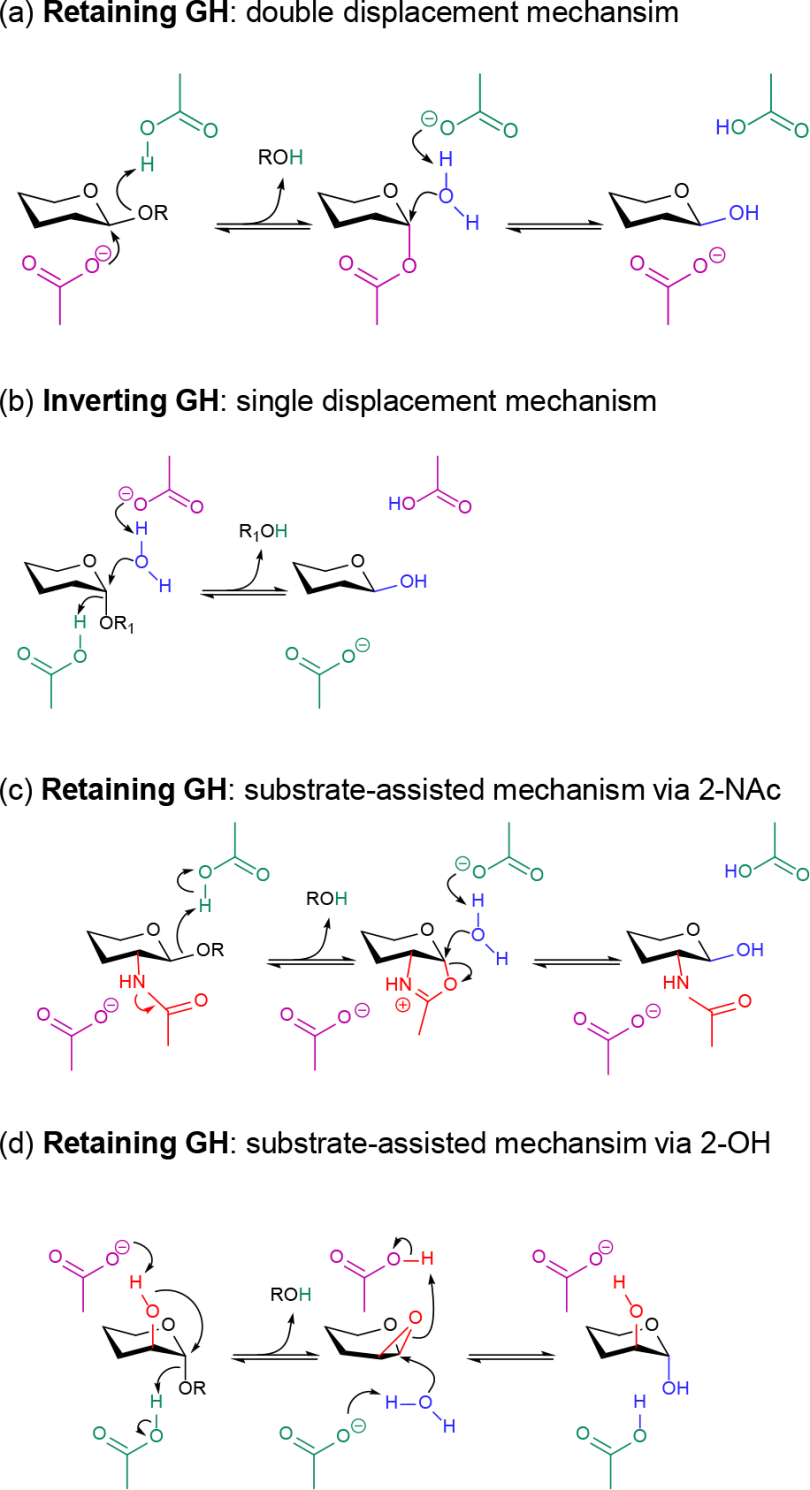
Mechanisms of Reactions Catalyzed by GHs

Such large diversity of structures needs a large number of enzymes responsible of their processing: formation, modification, and degradation. These are the so-called carbohydrate-active enzymes (CAZymes). There is a myriad of different types of CAZymes, phylogenetically organized in families,^4,5^ which exhibit a variety of structural folds, mechanisms, and specificities. The two most numerous groups are the glycoside hydrolases (GHs), which are responsible for the hydrolytic cleavage of carbohydrates (171 families to date), and the glycosyltransferases, which are responsible for the biosynthesis of carbohydrates (113 families to date).^4,5^ The understanding of CAZymes mechanisms has served to help to design mechanism-based inhibitors and to improve protein engineering for these enzymes. It is nowadays known that GHs follow not only the classical (Koshland) mechanisms^6^ of retention and inversion of the anomeric configuration (Scheme 1a,b, respectively), in which two carboxylic acid residues play the role of acid/base and general base residues (e.g., β-endoglucosidases^7−10^), but also other well-established mechanisms^11−13^ that depart from the classical ones. These include *substrate-assisted catalysis* (also called *neighboring group participation*), which can take place via the NAc substituent group at C2 (Scheme 1c) (e.g., families GH84 *O*-GlcNAcases and GH18 chitinases)^14,15^ or via the hydroxyl group at C2 (e.g., GH99 endo-β-mannanases)^16^ (Scheme 1d). Families GH4 and GH109 employ an oxidative mechanism,^11^ while GH172 operate through a retaining condensation mechanism involving a glycosyl-enzyme intermediate.^17^ Other unusual mechanisms have been recently proposed by theoretical methods.^18,19^ It is not obvious why numerous enzymes evolved to catalyze similar reactions with different mechanisms. In the case of the oxidative mechanisms, it seems that by using less efficient paths (multiple C–H bonds are formed and broken), the chemistry is shifted toward the glycoside rather than the aglycon departure. These mechanisms result in enzymes with low catalytic efficiencies but are able to act on both stereoisomers or even on *C*-glycosidic bonds.^20−23^ Mechanistic studies have not been limited to enzymes acting on pyranose-based substrates but have also been extended to furanose-based ones. Recently, retaining arabinofuranosidases of families GH51/GH54 and GH127/GH146, as well as inverting GH43 arabinofuranosidases, have been investigated with quantum mechanics/molecular mechanics (QM/MM) methods,^24−26^ providing insights to develop chemical probes and inhibitors targeting both α- and β-active enzymes.

**Scheme 2:**
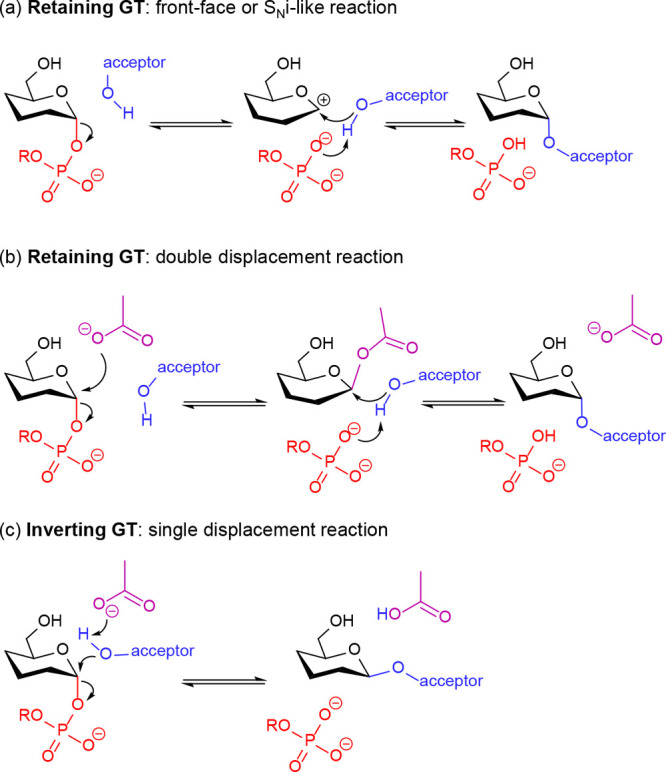
Mechanisms of Reactions Catalyzed by GTs

The mechanisms of GTs are less clear-cut than those of GHs. Most retaining GTs follow a *front-face* (also named as *S*_*N*_*i-like*) mechanism (Scheme 2a), in which a short-lived oxocarbenium ion-like intermediate is formed, although sometimes it is difficult to characterize it by theoretical methods.^27,28^ A number of retaining GTs that follow a front-face mechanism have been investigated in the last ten years by QM/MM approaches, such as thehalose-6-phosphate synthase (OtsA; family GT20),^29^ lipopolysaccharyl-α-1,4-galactosyltransferase C (lgtC; family GT8),^30^ polypeptide *N*-acetylgalactosaminyltransferase 2 (GalNAC-T2; family GT27),^31−33^ α-1,4-*N*-acetylhexosaminyltransferase (EXTL2; family GT64), glucosyl-3-phosphoglycerate synthase (GpgS; family GT81),^34^ α-1,3-xylosyltransferase (XXYLT1; family GT8),^35a^ glycogenin (GYG, GT8),^35b^ and mannosylglycerate synthase (MGS; family GT78).^36^ One mechanistic exception are the family GT6 enzymes, such as mammalian α-1,3-galactosyltransferase (α3GalT) and blood-group A and B α-1,3-glycosyltransferases (GTA/GTB), which can catalyze the formation of the glycosidic bond via a double-displacement mechanism (Scheme 2b).^37,38^ Unlike other GT families described so far, the active site of GT6 enzymes contains a catalytic nucleophile (glutamate) that is able to attack the anomeric carbon of the donor sugar; thus, the reaction can be completed in two steps, similarly to retaining GHs. Concerning inverting GTs, they are expected to follow a one-step S_N_2 reaction (Scheme 2c), similar to inverting GHs, such as the recently characterized *N*-acetylglucosaminyltransferase V (GnT-V; family GT18),^39,40^ but unconventional mechanisms such as asparagine tautomerization (in protein *O*-fucosyltransferase 1, POFUT1; family GT65)^41^ or substrate-assisted catalysis (in *O*-GlcNAc glycosyltransferase, OGT; family GT41)^42^ have also been proposed.

## CAZyme Mechanism Engineering

Understanding the mechanisms of GHs and GTs has paved the way for engineering of novel and/or enhanced functions in these enzymes.^43^ This, in turn, has attracted the industrial interest to offer enzyme-catalyzed solutions that meet the demands for eco-friendly processes.^44,45^ A variety of approaches that preserve the enzyme’s mechanisms are used, such as directed evolution,^46,47^ motifs exchange with related enzymes,^48−50^ active site systematic targeting,^51−53^ and structure-guided acceptor subsite targeting.^54−56^

**Scheme 3:**
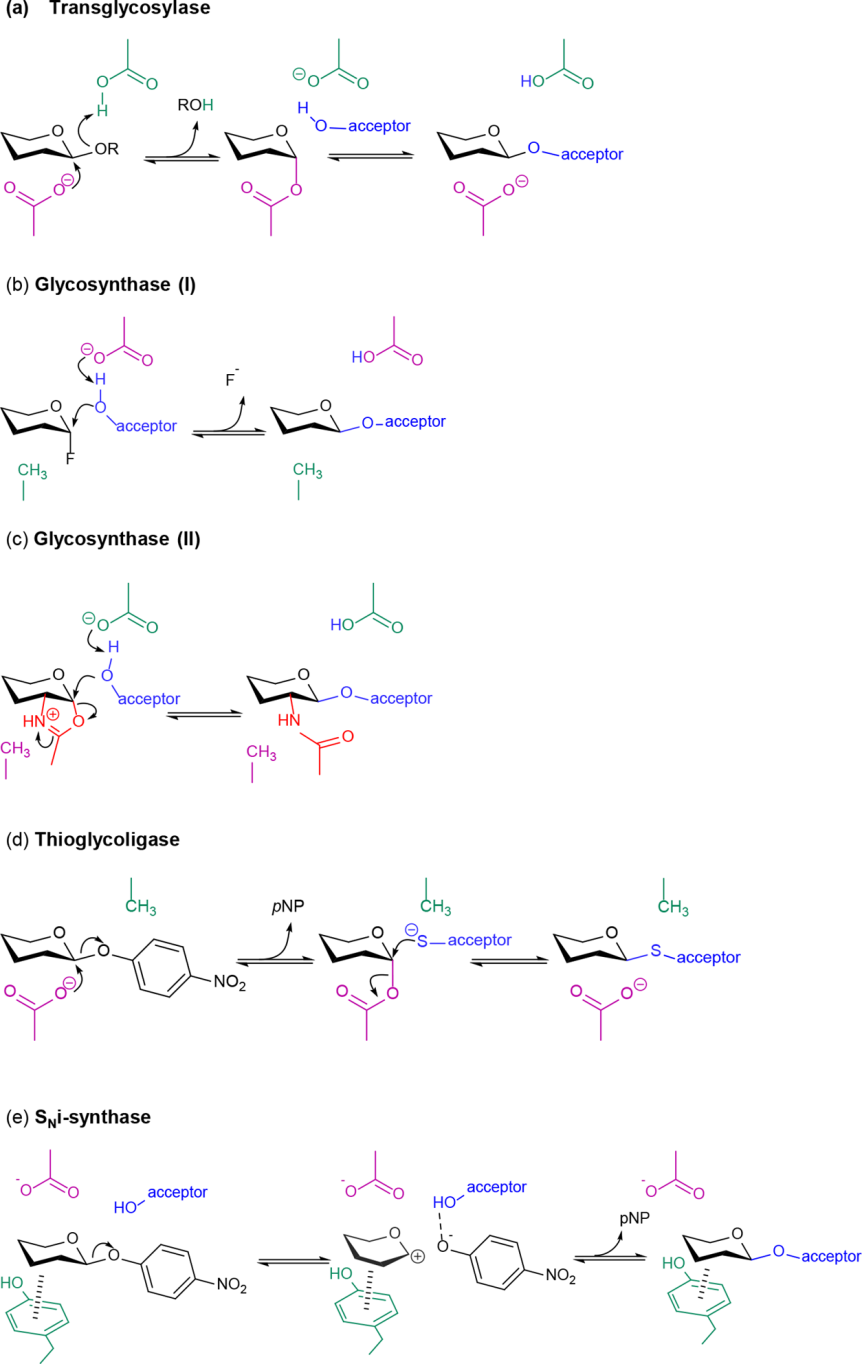
Mechanisms Catalyzed by Transglycosylases and Rationally Engineered GHs

Retaining GHs can be engineered for synthetic purposes by limiting their innate hydrolytic activity to favor their — usually secondary — transglycosylation activity. In this case, a carbohydrate acceptor rather than a water molecule reacts with the enzyme during the second reaction step (Scheme 3a).^57,58^ Several strategies have been tested for this purpose, and enzyme variants have been developed that significantly increase the synthetic yields,^54,59,60^ but the most successful engineering strategy to date for GHs is the glycosynthase approach (Scheme 3b),^61,62^ a rational design approach based on the modification of the enzyme mechanism combined with the use of artificial substrates. In glycosynthases, the nucleophilic residue of a retaining GH is mutated into a catalytically impotent residue, while the mutant enzyme is supplied with an activated donor residue of opposite anomeric configuration compared to the native enzyme. Experiments on glycosynthases from an *Agrobacterium sp*. β-glucosidase have shown that nucleophile mutation to Gly is the most efficient, followed by Ser and Ala; none of the other 16 possible permutations resulted in an effective glycosynthase.^61,63,64^ As the original catalytic nucleophile is absent, glycosynthases cannot hydrolyze the products they form, preventing secondary hydrolysis. Originally used on β-GHs along with α-glycosyl fluorides as substrate donors,^61,62^ this approach has since expanded to GHs operating via a substrate-assisted mechanisms combined with their bicyclic oxazoline intermediates^45,65^ as well as to α-GHs using β-azido glycosides (Scheme 3c).^66,67^ Interestingly, α-glucosyl fluorides have also recently been used to probe the mechanisms of both α (GH15) and β (GH55) inverting GHs, using a combination of secondary kinetic isotopic effects and QM/MM simulations, showing that in the case of fluoride departure, very little contribution from the incoming nucleophile is observed.^68^ The thioglycoligase variation (Scheme 3d) uses instead an acid/base mutant combined with activated acceptors and donors to form α- and β-thioglycosidic bonds^69,70^ and has since expanded both its donor and acceptor scope.^71,72^ While the glycosynthase approach is a one-step reaction with inversion of configuration (from α to β) (Scheme 3b), Davis and co-workers achieved synthesis with β-retention of configuration in one catalytic step (Scheme 3e).^73^ By means of appropriate mutation of the catalytic nucleophile and using glycoside substrates having aromatic leaving groups (e.g., para-nitrophenyl) that can form sugar-π and π–π interactions,^74,75^ the two substrate molecules accommodate in the active site in a configuration favorable for a S_N_i-like reaction that is similar to the one catalyzed by retaining GTs (Scheme 2a).^29^

The engineering of GTs is less developed compared to GHs. However, structure-based rational design has been quite successful in obtaining mutants with desired functionalities.^28^ For instance, family GT43 (inverting GTs responsible for the synthesis of the tetrasaccharide core structure of glycosaminoglycans such as heparan sulfate, heparin, chondroitin sulfate, and dermatan sulfate in proteoglycans) have been engineered by mutation of a conserved site histidine to arginine, making them more promiscuous against different sugar-nucleotide donors.^76^ Human blood group A and B GTs, which are family GT6 enzymes responsible for the formation of terminal glycosidic linkages of blood group antigens, have been engineered to introduce residues from one blood type GT into the other, leading to changes in substrate specificity and catalytic activity.^77,79^ Recently, family GT1 enzymes have been engineered to tune their substrate specificity,^78^ as discussed in detail later on.

## Computer Simulation of CAZyme Mechanism Engineering

Computer simulation is nowadays a great aid to rational CAZyme engineering, as simulations can interpret and even predict reaction outcomes. A prerequisite to modeling CAZymes is to adequately choose the type of model (e.g., the full enzyme) and the level of theory (e.g., *ab initio* or semiempirical methods for the QM region) needed to describe the process of interest (e.g., chemical reaction or substrate binding/unbinding), both related to the time scale and to the specific biochemical event under investigation. QM/MM simulations combined with MD (QM/MM MD) represent a good compromise among accuracy, resolution, and computational cost, providing a detailed view of processes in which chemical bonds are formed or broken. If they are used along with enhanced-sampling methods (or another free energy method), enzyme reactions can be modeled, providing in-depth understanding of how changes in the enzyme active site affect enzymatic mechanisms. When more than one reaction can happen, simulations allow identifying the most favored one and understanding what discriminates among them. Simulations also allow characterization of reactions that are not (yet) observed experimentally and provide insight into other aspects of catalysis such as the binding of substrates^59,80,81^ and the unbinding of reaction products.^82^ In the last cases, no relevant electronic reorganization takes place; thus a force-field description of the interactions among atoms, such as in classical MD and docking approaches, are typically the method of choice.

The above considerations are exemplified with two of our recent studies in which rational design was applied to modify the function of glycoprocessing enzymes (1) to convert a GH into a glycoside phosphorylase and (2) to engineer the specificity of a glycosyltransferase that catalyzes the formation of *O*-glycosidic bonds toward the formation of *N*- or *S*-glycosidic bonds. In both cases chemical intuition was not enough to fully explain the experimental data; thus, computer simulations were used to achieve this milestone, a necessary step to further engineer new variants.

## From Glycosidases to Phosphorylases: Turning Hydrolysis into Phosphorylation

Family GH84 *O*-GlcNAcases play an essential role in biology, as they are responsible for one of the main reactions involved in protein *O*-glycosylation: the cleavage of the *N*-acetylglucosamine (GlcNAc, a glucose derivative) that is attached to a Ser or Thr residue in proteins.^83^ Dysregulation of *O*-GlcNAcylation and *O*-GlcNAc cycling enzymes has been detected in many diseases including cancer, diabetes, cardiovascular, and neurodegenerative diseases, thus making *O*-GlcNAc enzymes of high biomedical relevance.

Given their crucial role in biology, GH84 *O*-GlcNAcases have been extensively studied.^14,84−87^ From a mechanistic point of view, GH84 *O*-GlcNAcases follow the substrate-assisted mechanism depicted in Scheme 1c,^14,88,89^ operated by two Asp residues. In the first reaction step, one Asp takes a proton from the substrate *N*-acetamido group (NAc), while the oxygen atom of NAc attacks the anomeric carbon to form an oxazolinium ion reaction intermediate.^87^ The other catalytic Asp acts as a general acid/base, assisting leaving group departure by protonating the glycosidic bond oxygen.^14,84−87^ In the second step, a water molecule, activated by the general acid/base residue, attacks on the anomeric carbon to release the product, GlcNAc.

**Figure 1:**
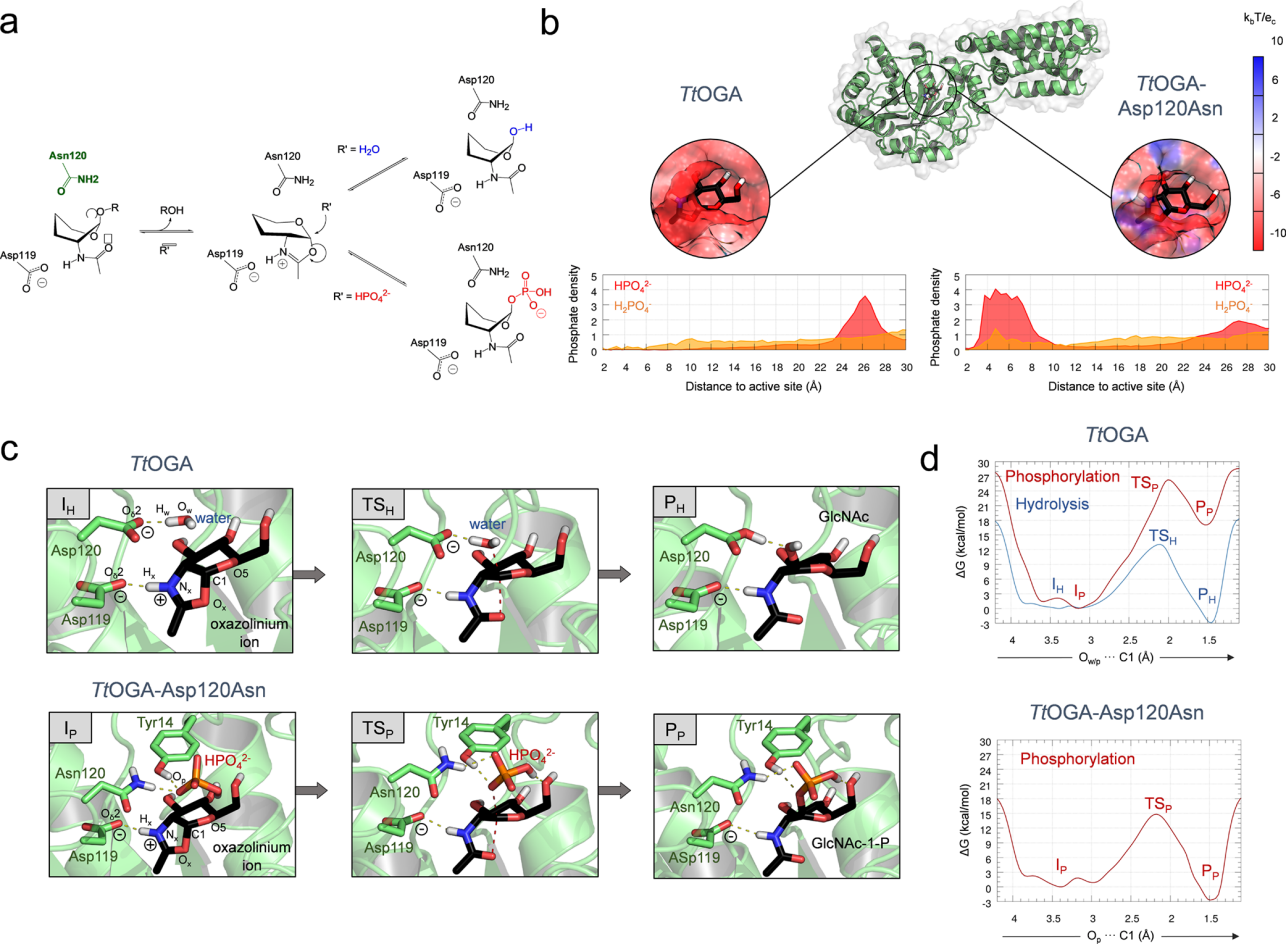
(a) Hydrolysis and phosphorylation reactions catalyzed by OGA upon mutation of the acid/base residue (Asp120 in *Tt*OGA) to Asn. (b) Computed electrostatic potential of *Tt*OGA at the active site and radial distribution function of the distance between the Cγ of the Asp120/Asn120 residue and the closest oxygen of each phosphate ion. (c) Representative structures along the reaction coordinate for *Tt*OGA hydrolysis and *Tt*OGA-Asp120Asn phosphorolysis. I_H_, TS_H_, P_H_ = intermediate, transition state, and products of the hydrolysis reaction, respectively. I_P_, TS_P_, P_P_ = intermediate, transition state, and products of the phosphorylation reaction, respectively. Bonds being formed/broken are indicated with red dashed lines. (d) Free energy profile of the reactions catalyzed by for *Tt*OGA and *Tt*OGA-Asp120Asn. Reproduced from ref (90). Copyright 2020 American Chemical Society.

Recently, two bacterial GH84 *O*-GlcNAcases (from *Streptococcus pyogenes* and *Thermobaculum terrenum*) were converted into synthetic phosphorylases to produce phosphorylated carbohydrates, highly valuable compounds, by mutation of one active site residue.^90^ In particular, the Asp acid/base residue was substituted by an Asn. This was a sought-after conversion inspired by the naturally occurring GH3 enzymes, which can be either hydrolases or phosphorylases depending on the acid/base active site residue (Asp in GH3 hydrolases and His in GH3 phosphorylases). However, mutation of the native Asp acid/base residue to His in GH84 *O*-GlcNAcases severely reduced hydrolysis and did not enable this variant to catalyze phosphorylation. Instead, mutation of the same residue for an asparagine (Asp→Asn) drastically changed enzyme activity from hydrolysis to phosphorolysis (Figure 1a). There was no obvious explanation for such novel conversion; therefore, we turned to computer simulation to “rationalize” the results of the rational design.

By means of computer simulations of the reaction intermediate, which is the common species in the mechanism of hydrolysis and phosphorylation, we analyzed several factors that may play a role in catalysis.^90^ We performed classical molecular dynamics (MD) on wild-type OGA from *Thermobaculum terrenum* (*Tt*OGA) and its Asp120Asn variant (*Tt*OGA-Asp120Asn). A glucose oxazolinium ion was bound in the active site of both enzymes, mimicking the intermediate species of the reaction. The model included the experimental concentration of phosphate ions (20 mM at pH 7.8), since that was the only source of phosphate groups in the experimental setup. The MD simulations showed that the distribution of phosphate anions was completely different when the wild-type enzyme and the Asp120Asn variant were compared: phosphates were unable to visit the vicinity of the wild-type active site but instead reached and populated the active site of the Asp120Asn variant (Figure 1b). The reason behind this observation was attributed to changes in the electrostatics of the active site upon mutation. Substitution of Asp120 by an Asn reduces the negative charge, allowing phosphate anions to come close. This is reflected in the drastic change of the active site electrostatic potential, from negative in the native enzyme to positive in the case of the engineered *Tt*OGA-Asp120Asn variant (Figure 1b). Therefore, mutating the Asp acid/base residue to Asn diminishes the electrostatic repulsion with phosphate anions and thus allows them to approach and explore favorable positions for nucleophilic attack.

The computed reaction mechanisms of hydrolysis and phosphorylation calculated by QM/MM metadynamics shed further light on the molecular determinants that discriminate between both reactions. The hydrolysis reaction by the wild-type enzyme consists in a S_N_2 reaction in which a water molecule, deprotonated by the acid/base residue, performs a nucleophilic attack onto the anomeric carbon of the glucose oxazolinium ion substrate (Figure 1c). The reaction turns to be exergonic (the GlcNAc product is more stable than the reactant) and the free energy barrier (13.2 kcal/mol) is in good agreement with experiments, Δ*G*^‡^ = 14.7 kcal/mol) (Figure 1d). On the contrary, phosphorylation by the wild-type enzyme led to an endergonic reaction with a significantly higher free energy barrier (Δ*G*^‡^ = 26.3 kcal/mol). This is consistent with the experimental observation that the wild-type enzyme does not catalyze phosphorolysis. Even though the reaction mechanism is similar to that of hydrolysis (i.e., the nucleophile molecule is deprotonated by the acid/base and attacks the anomeric carbon), phosphorylation is neither thermodynamically nor kinetically favored. Therefore, the simulations reveal that, even if a phosphate anion approaches the wild-type active site, which does not occur according to our classical MD calculations, it would not react.

In the same vein, we analyzed phosphorylation of the glucose oxazolinium ion by the Asp120Asn variant. In this case, the computed reaction mechanism is exergonic with a free energy barrier of 14.8 kcal/mol, consistent with experimental data (Δ*G*^‡^ = 16.3 kcal/mol). When the atomic rearrangements of the mutant active site during the enzymatic reaction were analyzed, other factors appeared to be crucial for phosphorylation. The introduced Asn residue not only forms a hydrogen bond with the phosphate nucleophile but also places a nearby tyrosine (Tyr14) in a configuration in which it is also able to form a hydrogen bond to the phosphate. Both hydrogen bonds stabilize the incoming phosphate anion as well as the reaction products (GlcNAc-P). In the case of the wild-type enzyme, Tyr14 cannot act as a hydrogen bond donor for phosphate ions, as it is interacting with the carboxylate group of Asp120 (acid/base residue). This highlights how a small perturbation of hydrogen bond networks can drastically affect catalysis.

In conclusion, computer calculations revealed that introduction of an Asn in the active site of GH84 *O*-GlcNAcases provides an optimal catalytic machinery to accommodate negatively charged phosphate ions and use them to phosphorylate carbohydrates. The insight gained in this work can inspire further experiments to synthesize high-value phosphorylated sugars. Interestingly, a recent study of a GH3 xylosidase revealed that mutation of the acid/base Glu residue to Ala is also able to catalyze phosphorolysis among numerous ligase reactions.^91^ This shows that the isosteric replacement is not necessarily the best solution, yet the molecular understanding of why the Ala variant outperforms other mutations remains unclear.

## *À la Carte* Formation of *O*-/*N*-/*S*-Glycosidic Bonds by an Engineered Glycosyltransferase

Glycosyltransferases from family GT1 are particularly interesting for their ability to catalyze the formation of several types of glycosidic bonds. These enzymes act on a vast array of acceptors in plants, animals and bacteria. Bifunctional *O*-/*N*-^101−103^ and *O*-/*S*-transferases^104^ have been reported, as well as trifunctional *O*-/*N*-/*S*-transferases.^105,106^ While no enzyme is known to form a *C*-glycosidic bond on a sp^3^ carbon, some GTs can also catalyze the formation of *C*-glycosidic bonds with an aryl moiety, most likely via an electrophilic aromatic substitution (S_E_Ar) mechanism, while still catalyzing *O*-glycosylation via a S_N_2.^92,93^ Yet, the limited number of structures of GT1 enzymes in complex with donors and/or acceptors hinders dissecting their mechanism of action in detail. In addition, a direct kinetic comparison on structurally similar acceptors is not available, thus the determinants of the enzyme’s specificity for R-OH, R-NH_2_, and R-SH substrates is poorly understood.

**Figure 2:**
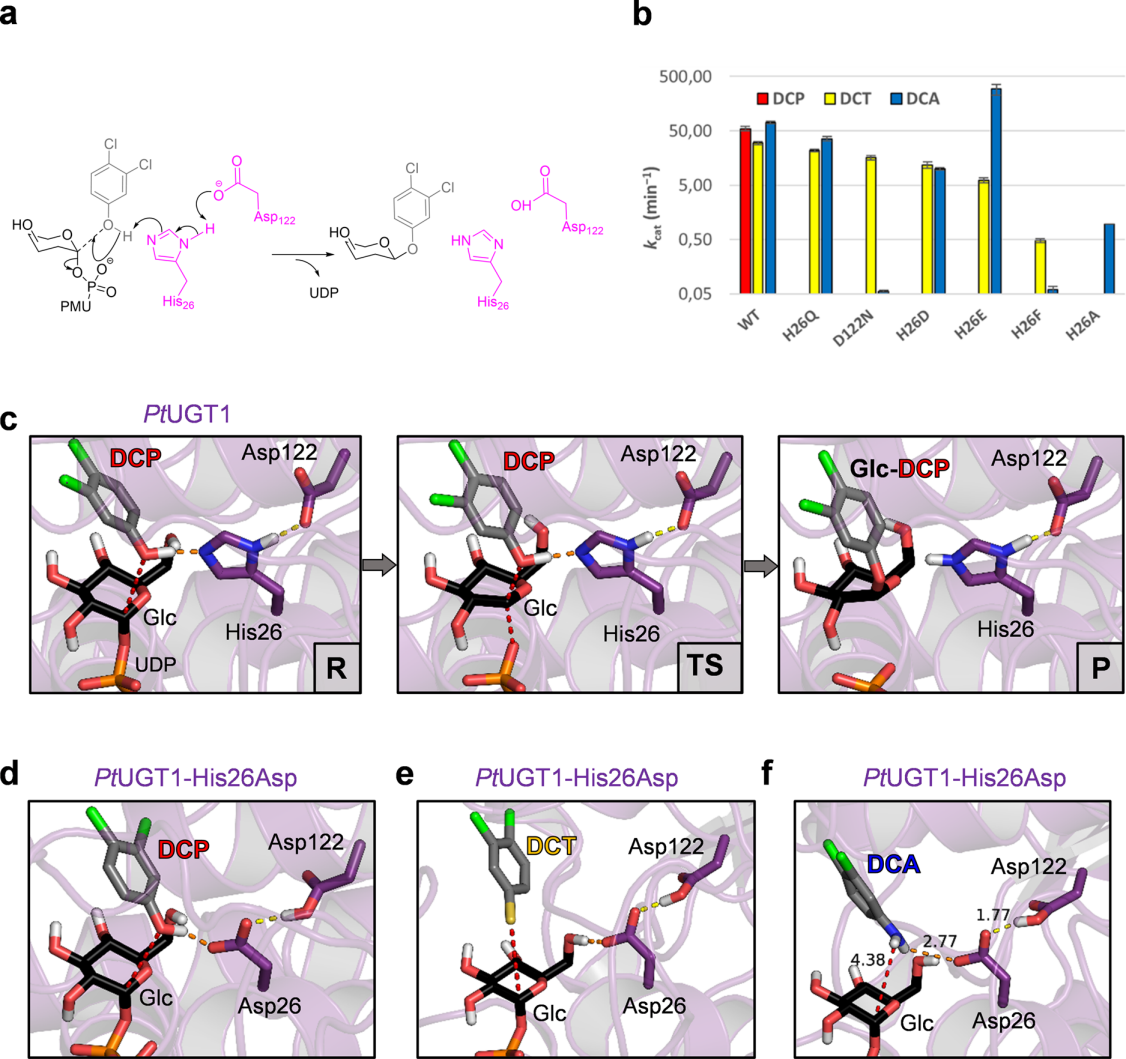
(a) Activities of *Pt*UGT1 variants at pH = 7 in the presence of 1 mM UDP-Glc and 500 μM acceptor. *k*_cat_ toward DCA (blue), DCP (red), and DCT (yellow) are shown on a logarithmic scale. (b) *O*-glycosylation reaction catalyzed by *Pt*UGT1 with the DCP acceptor. (c) Mechanism of the *O*-glycosylation reaction computed by QM/MM metadynamics. (d, e, f) Michaelis complexes of *Pt*UGT1-His26Asp with DCP, DCT, and DCA, respectively, obtained from QM/MM MD (DCP and DCT) or MD simulations (DCA). Reproduced from ref (78). Copyright 2021 American Chemical Society.

In a recent study, the specificity of a trifunctional *O*-/*N*-/*S*-GT1 enzyme was tuned to efficiently and selectively form only either C–N or C–S bonds.^78^ The system investigated is a GT1 enzyme from *Polygonum tinctorium* (*Pt*UGT1) that operates with inversion of the anomeric configuration (Scheme 2c (Figure 2a). The native mechanism consists of a single S_N_2 reaction in which His26 (activated by Asp122) deprotonates the incoming acceptor, which performs nucleophilic attack on the sugar anomeric carbon, releasing UDP. In our investigation, 3,4-dichloro aromatic acceptors were used to evaluate *O*-/*N*-/*S*-specificity (3,4-dichlorophenol, 3,4-dichloroaniline, and 3,4-dichlorothiophenol, respectively). For convenience, these are hereafter named DCP, DCA and DCT, respectively.

Mutation of the catalytic dyad residues (His26 and Asp122) produced variants with different relative *O*-, *N*-, and *S*-glycosylation activities, thus altering the enzyme specificity for a given substrate (Figure 2b). While monospecific *N*- and *S*-glycosylation was achieved by *Pt*UGT1-His26Ala and *Pt*UGT1-Asp122Asn, respectively, enhanced *N*-glycosylation compared with the wild-type enzyme was enabled by *Pt*UGT1-His26Glu. An appropriate mutation (His26 to Gln) knocked out *O*-glycosylation while maintaining high rates for the other two reactions.

Computer simulations were used to rationalize the experimental observations and determine the molecular basis of *O*-/*N*-/*S*-glycosylation in *Pt*UGT1. Classical molecular dynamics (MD) simulations and QM/MM MD simulations were performed on several variants. The first gave information whether a certain variant can accommodate a specific acceptor in the active site conformation, whereas the latter confirmed that the complex can effectively react. For instance, MD simulations of *Pt*UGT1-His26Phe in complex with DCA (*N*-acceptor) showed that the introduced phenylalanine residue sits between the acceptor and the donor molecules, precluding them from being close enough to react. This is consistent with the lack of activity that this variant exhibits toward the anilinic substrate (DCA). However, MD simulations alone were not able to explain the observed *O*-/*S*-specificity differences. In particular, why DCP (*O*-acceptor) only reacts in the wild-type enzyme, while DCT (*S*-acceptor) is also catalytically active when His26 is mutated to either Asp or Glu.

To rationalize the above observations, QM/MM metadynamics calculations were performed on the wild-type enzyme in complex with DCP (Figure 2c), as well as the His26Asp mutant in complex with DCP and DCT (Figure 2d, e). The simulations of the reaction of DCP with *Pt*UGT1 and *Pt*UGT1-His26Asp showed that only the native His26 is able to maintain a reactive conformation in which Asp122 activates the catalytic base. Asp26 in this position cannot interact with Asp122, which misorients the DCP substrate and makes the S_N_2 attack difficult. Consistently, a high reaction free energy barrier (39 kcal/mol) was observed for the reaction of *Pt*UGT1-His26Asp with DCP. This is not the case for the wild-type enzyme, where the catalytic dyad His26-Asp122 enables optimal deprotonation of DCP by His26 and its nucleophilic attack on the donor sugar (Figure 2c), resulting in a much lower free energy barrier of (17.2 kcal/mol), in very good agreement with the experimental estimation (17.6 kcal/mol).

*S*-Glycosylation differs from *O*-glycosylation in that the DCT acceptor (p*K*_a_ = 5.47) is expected to react in its thiophenolate form; thus, it does not require deprotonation. The structure of the Michaelis complex of *Pt*UGT1-His26Asp with DCT (Figure 2e) obtained by QM/MM MD simulations revealed that DCT is perfectly poised for nucleophilic attack on the anomeric carbon of the glucosyl donor. Additionally, the negative charge on the thiophenolate group further stabilizes the positive charge being developed on the anomeric carbon at the reaction transition state. Hence, there is no direct involvement of residue 26 in the catalysis (Figure 2e), which effectively takes place without the participation of any catalytic residue. This explains the limited effects of His26 mutations on *S*-glycosylation.

The overall results for the three acceptors indicate that substrate selectivity in *Pt*UGT1 is governed by structural and electrostatic factors: *O*-glycosylation requires the native His-Asp dyad to catalyze the nucleophilic attack via acceptor deprotonation by His26. In contrast, both *N*- and *S*-glycosylation can happen without proton transfer with a catalytic residue and depend on the acceptor positioning relative to the donor. Altogether, we elucidated the structural and electronic features that govern substrate selectivity by *Pt*UGT1, which would facilitate further GT engineering efforts to effectively form *O*-/*N*-/*S*-glycosidic bonds.

## Final Remarks

Collaboration between experimentalists and modelers is important to push forward enzyme engineering investigations. While enzyme variants are produced and tested in the laboratory, and usually designed on the basis of rational approaches, molecular simulations can assist in predicting and further understanding the details of the molecular mechanisms. Particularly, simulations allow testing reactions that are not observed experimentally, rationalizing how enzymes discriminate among several candidate reactions and understanding why some reactions do not happen. Here we reviewed the recent literature on rational engineering of CAZymes and summarized two investigations in which the initial rational design was not enough to understand the experimental data. In both cases, classical MD and QM/MM MD were employed. The first was used to model Michaelis complexes of CAZymes with natural or artificial substrates, with the aim of predicting whether they could be reactive, whereas the latter were used to model the chemical reaction itself, confirming that it is plausible. The molecular insights into the reactions catalyzed by both wild-type and engineered enzymes allowed us to rationalize “rational” engineering of these CAZymes.
